# Heard and valued: the development of a model to meaningfully engage marginalized populations in health services planning

**DOI:** 10.1186/s12913-018-2969-1

**Published:** 2018-03-15

**Authors:** M. Elizabeth Snow, Katherine Tweedie, Ann Pederson

**Affiliations:** 1 Centre for Health Evaluation & Outcome Sciences (CHÉOS), University of British Columbia, St. Paul’s Hospital, 588 - 1081 Burrard Street, Vancouver, BC V6Z 1Y6 Canada; 2 University of British Columbia, 2329 West Mall, Vancouver, BC V6T 1Z4 Canada; 3 BC Women’s Hospital + Health Centre, E305 - 4500 Oak Street, Vancouver, BC V6H 3N1 Canada

**Keywords:** Patient engagement, Healthcare planning, Marginalized populations, Equity, Gender

## Abstract

**Background:**

Recently, patient engagement has been identified as a promising strategy for supporting healthcare planning. However, the context and structure of universalistic, “one-size-fits-all” approaches often used for patient engagement may not enable diverse patients to participate in decision-making about programs intended to meet their needs. Specifically, standard patient engagement approaches are gender-blind and might not facilitate the engagement of those marginalized by, for example, substance use, low income, experiences of violence, homelessness, and/or mental health challenges—highly gendered health and social experiences. The project’s purpose was to develop a heuristic model to assist planners to engage patients who are not traditionally included in healthcare planning.

**Methods:**

Using a qualitative research approach, we reviewed literature and conducted interviews with patients and healthcare planners regarding engaging marginalized populations in health services planning. From these inputs, we created a model and planning manual to assist healthcare planners to engage marginalized patients in health services planning, which we piloted in two clinical programs undergoing health services design. The findings from the pilots were used to refine the model.

**Results:**

The analysis of the interviews and literature identified power and gender as barriers to participation, and generated suggestions to support diverse populations both to attend patient engagement events and to participate meaningfully. Engaging marginalized populations cannot be reduced to a single defined process, but instead needs to be understood as an iterative process of *fitting* engagement methods to a particular situation. Underlying this process are principles for meaningfully engaging marginalized people in healthcare planning.

**Conclusion:**

A one-size-fits-all approach to patient engagement is not appropriate given patients’ diverse barriers to meaningful participation in healthcare planning. Instead, planners need a repertoire of skills and strategies to align the purpose of engagement with the capacities and needs of patient participants. Just as services need to meet diverse patients’ needs, so too must patient engagement experiences.

**Electronic supplementary material:**

The online version of this article (10.1186/s12913-018-2969-1) contains supplementary material, which is available to authorized users.

## Background

Recently patient engagement has been identified as a promising strategy for supporting health services planning by health organizations in British Columbia [1, 2], Canada [3–5], and internationally [6–8]. Patient engagement, in this context, refers to processes through which patients’ values, needs, and preferences inform health services planning with the intended goal of making health services more accessible, appropriate and acceptable to patients [14]. We believe that this process does not bring health services closer to patients’ needs unless the process is meaningful to the patients, meaning patients’ values, preferences, and needs are heard, understood, and valued by health service decision makers and actually affect the services and policies being developed.

Often the context and structure of patient engagement processes may systematically exclude marginalized populations from participating in a meaningful way or participating at all. Most patient engagement processes consist of “activating” patients, i.e., teaching them to participate in healthcare discussions and then inserting them into a healthcare planning process to provide a “patient” perspective. This approach (and other traditionally-used formats of patient engagement, such as advisory councils and deliberative processes) privileges participation styles similar to those of health service planners. Unfortunately, these processes may exclude groups of patients, such as women who are low-income, sexual minorities, and sex workers, who face various barriers to health services, yet may have poor mental and physical health and make frequent use of healthcare. Universalistic, “one-size-fits-all” approaches may not enable diverse patients to participate in decision-making about programs and policies intended to meet their needs or enable planners to develop interventions with diverse patients.

We came to this work with a commitment to reducing health inequities, many of which arise from the very conditions that marginalize and disempower some women in Canada [6, 9]. The goal of this project was to develop a model to meaningfully engage women in health services planning, with a focus on including a diversity of women whose voices are typically excluded from such processes. As researchers and program developers in women’s health, we were concerned that standard patient engagement approaches were gender-blind [10, 11], assuming gender does not influence participation in, or results of, engagement [12, 13]. Therefore, we felt such processes might not facilitate engagement of women, particularly those who were marginalized by issues such as substance use, low income, experiences of violence, homelessness, and/or mental health challenges.

Using a qualitative research approach, we reviewed literature and conducted interviews with self-identified female community members and with health services planners regarding engaging marginalized populations in health services planning. We used these initial inputs to create a model for engaging patients and a planning manual which we then piloted in two clinical programs undergoing health services planning. We refined the model and revised the planning manual based on the experiences and findings from the pilot projects and created an online learning module to disseminate these tools.

## Methods

### Phase I

#### Literature review

We reviewed reports of patient engagement initiatives that explicitly sought to engage diverse or vulnerable populations to describe challenges and strategies to meaningfully engaging marginalized populations. Abelson et al.’s three criteria were used to select initiatives for the review: 1) participants received information about an issue; 2) participants had opportunities to discuss the issue amongst themselves and potentially with decision makers; and 3) there was an explicit process for collecting participants’ input [14].

We searched for relevant articles in Cumulative Index to Nursing and Allied Health Literature (CINAHL) and Medical Literature Analysis and Retrieval System Online (MEDLINE®) using the terms: patient engagement, patient participation, user designed, community engagement, decision making; gender, women, marginalized, vulnerable, social justice, healthcare, health services, consumer participation, patient participation, vulnerable populations, women, disabled persons, drug users, emigrants and immigrants, homeless persons, sex workers, transients and migrants, program development, planning techniques, health services research, advisory committees, service(s), and program(s). We searched for additional articles and grey literature through reference lists of selected articles and a web search. The literature search was conducted in 2012 and updated in 2013. We did not use a historical date cutoff, yet we did not find relevant literature published prior to the 1990s. A total of 61 articles were identified.

Articles were included if the engagement initiative aimed to improve health services for populations who needed additional or unique health service supports and the engagement strategies sought to overcome exclusionary characteristics of traditional engagement processes. Articles were excluded if engagement referred to patients participating in their own healthcare; engagement was for the purpose of research instead of shaping a policy, program, or system; if the article did not describe the engagement process; or if the project took place in a developing country.

In total, 17 peer-reviewed articles were ultimately examined, supplemented by findings from a literature review on consumer participation and diversity performed by the National Resource Centre for Consumer Participation in Health in Australia [15]. No other grey literature meeting the inclusion criteria was found, likely due to insufficient incentives for health agencies to document and publicly share such processes, and barriers to making such documents available (e.g., privacy and permissions; few websites to host reports).

To identify themes, projects were categorized by level of engagement based on the International Association of Public Participation (IAP2) spectrum of public participation [11], and examined for trends in the purpose of engaging patients, strategies used to engage participants, and successes and challenges in their engagement of diverse and marginalized populations.

#### Focus groups and interviews

Concepts identified through the literature review were interrogated through 1) semi-structured interviews and focus groups with female community members to explore how they would want to participate in health services planning and how they would define effective patient engagement (see Additional file 1 for interview and focus group guides) and 2) semi-structured interviews with health service planners and researchers who had experience engaging patients, particularly people whose voices have not traditionally been heard in health services planning (see Additional file 2 for interview guides). Thirteen women were recruited through community-based agencies working with diverse populations, including women who were: pregnant and/or new parenting mothers affected by substance use and/or violence; new immigrants to Canada; Indigenous and/or had a history of incarceration. Nineteen health services planners, managers, coordinators, and people who facilitated patient engagement processes were recruited from a health authority, as well as contacts in other local agencies recommended by initial interviewees.

A grounded theory approach [16] was used to examine interview content and modify interview/focus group questions and processes in an iterative fashion. Although we original intended to interview more community members, through continual comparative analysis of the data we learned the concept of engaging in health services planning was very abstract and not necessarily perceived as relevant to community members’ experiences. Interviews with health service providers further confirmed that engaging patients regarding abstract healthcare concepts provides few opportunities for patients to contribute unless they do considerable work in preparation for participation. Thus, fewer women were interviewed than initially planned.

#### Data analysis

Our data analysis sought to identify strategies for planners to engage marginalized patients in a meaningful way, both pertaining to specific levels of engagement using the IAP2 Spectrum of Public Participation, as well as trends cutting across all levels of engagement.

As interviews were completed, they were transcribed verbatim and uploaded into QSR NVivo qualitative analysis software [17]. The coders [MES and KT] debriefed after interviews to discuss themes, compare emergent ideas against the emerging model, and identify and pursue emergent lines of inquiry. The coders independently coded the data using both a priori codes based on stage of the engagement process (e.g., recruiting participants) and emergent codes using a constant comparative approach. Subsequently, the two researchers compared codes; differences were discussed and resolved and they wrote memos to document the coding process.

#### Developing the model

**Fig. 1:**
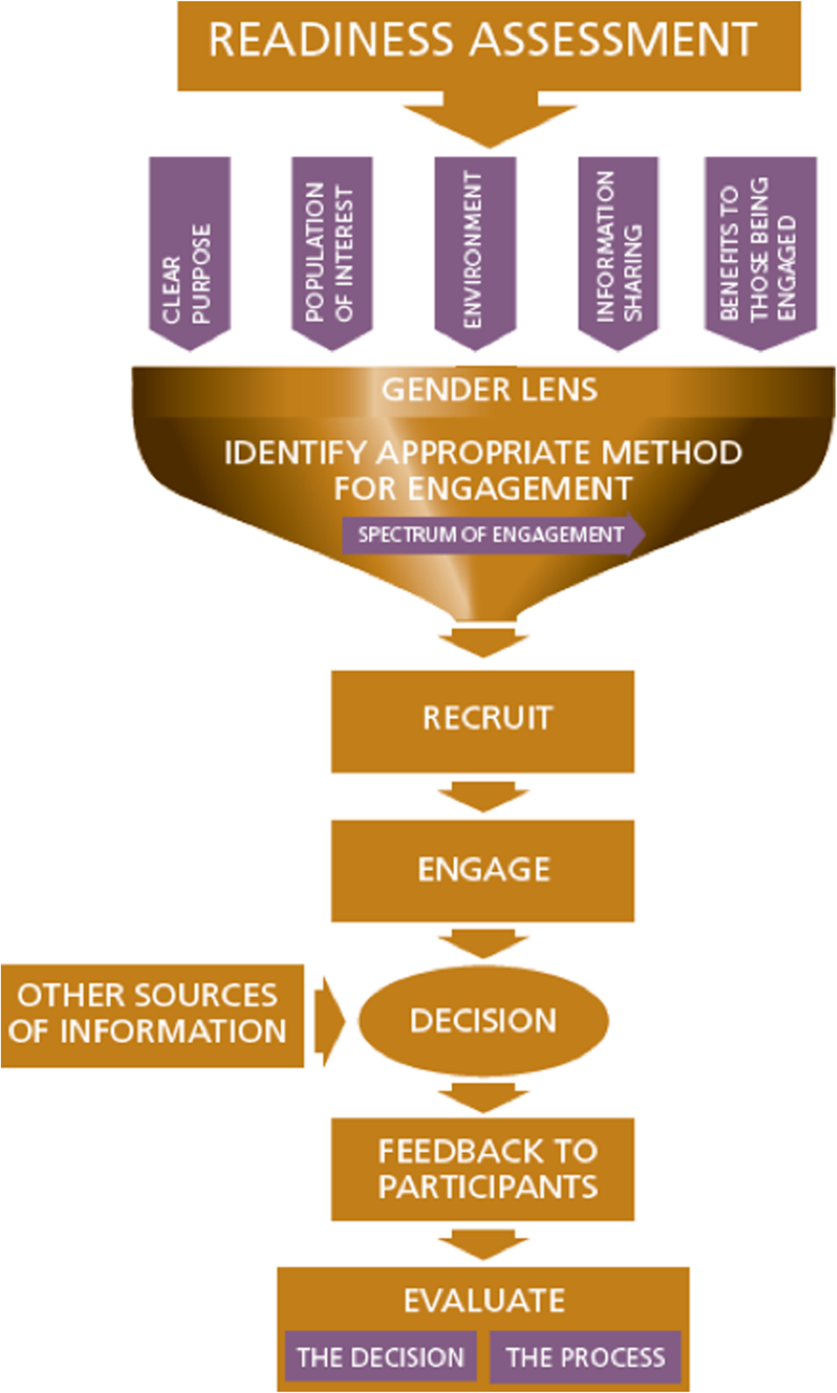
Initial model

The first iteration of the model (see Fig. 1) was developed by synthesizing the literature and themes from interviews/focus groups into a visual model to guide planning and implementing patient engagement. The emergent model identified systematic barriers and power dynamics embedded in patient engagement processes, as they relate to the social location of their particular population of interest. It was intended to guide planners to minimize these barriers and empower patients in the engagement process. Core elements included: a readiness assessment; strategies for identifying a suitable engagement method; planning engagement events; conducting gender-sensitive engagement; and evaluating the effect of engagement on planning. A planning manual was created to support planners to use the model.

The draft model was presented to researchers, policy-makers, and health services planners at three events. Although these presentations generated interest, participants offered little substantive feedback on the model. Patient participants also found it difficult to provide feedback. Indeed, patients interviewed indicated that “patient engagement” is an abstract concept disconnected to their healthcare experiences. Our findings suggest that asking patients to engage with such issues puts an additional burden on them to learn about the abstract concept, and may diminish the importance of patients’ values, preferences, and lived experience. Moreover, as the model is intended to put the onus on healthcare planners to learn more about issues of marginalization and to create an engagement process that is sensitive and appropriate to marginalized patients, we chose to not elicit feedback from patients on the model as we had originally planned.

Phase I of the project was originally intended to develop standards for evaluating patient engagement; however, few articles included evaluation of patient engagement interventions. Some evaluated process, such as people satisfaction with the engagement process. However, evaluations of outcomes of patient engagement (i.e., effect of patient engagement on actual health services or policy) were not found in the literature. Developing standards by which to evaluate without a concrete patient engagement project in mind also proved to be an abstract concept that was difficult to assess for the women we interviewed. However, at the most basic level, patients interviewed in Phase I noted they would consider patient engagement to be successful if something *changed* based on their input.

### Phase II: Piloting the model

**Fig. 2:**
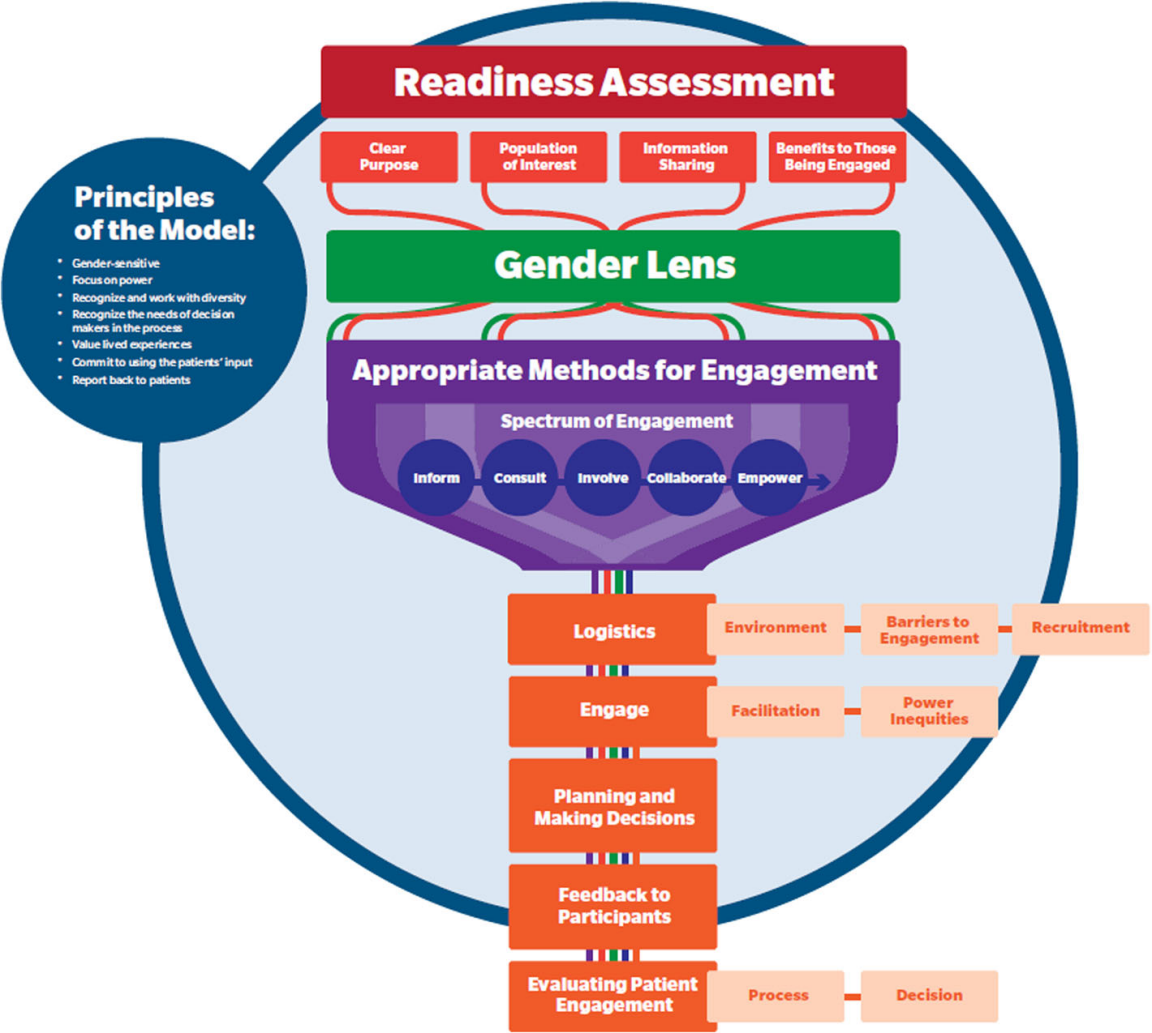
Final model

The model was piloted in two clinical programs. A Public Health program used the model to engage 70 patients to inform service planning of nurses providing home visits to pregnant and new parenting mothers in vulnerable families. A primary care program used the model to engage 9 patients in the redesign of a chronic disease education program provided in both English and Punjabi. In both pilots we worked with planners from clinical programs to apply the model to their programs, and observed which elements of the model and planning manual worked well and what was unclear, and noted suggestions for improvement. We developed a second iteration of the model based on the experiences of the two pilot projects (see Fig. 2).

#### Research ethics

This project received research ethics approval from the Fraser Health Authority Research Ethics Board. Participants in Phase I interviews and focus groups and Phase II patient engagement sessions signed informed consent forms. Identities of participants were kept confidential, with only the research team having access to audio recordings and transcripts.

## Results

### Literature review findings

Table 1 summarizes findings of the literature review by level of engagement from the IAP2 Spectrum of Public Participation [11] and three aspects of engagement: purpose of engaging; characteristics of the participants they succeeded in engaging; and strategies used to engage participants. The “inform” level of engagement from the IAP2 spectrum was excluded because it does not meet Ableson et al.’s [14] three criteria for engagement in decision making (i.e., participants did not have opportunity to discuss the issue nor is input collected from participants in the “inform” level).

**Table 1:** Summary of Literature Review Findings by Level of Engagement

Category of Engagement (IAP2)	Purpose of Engaging	Engagement Participants	Engagement Strategies	References
Consult	▪ Identify system-level healthcare gaps and barriers experienced by participants ▪ Collect participants’ ideas for improving services/policies ▪ Inform development of evaluation, collect evaluation data	▪ Users of various healthcare system from diverse backgrounds ▪ Users of specific community-based services	▪ 1-day in-person events ▪ Participants shared health experiences with each other through various discussion formats (e.g. world café, interpersonal conversations, writing and drawing on poster boards) ▪ Solicited ideas by asking what the system is currently like, and how it should ideally be ▪ Participants shared ideas about how healthcare could be made better	[15, 18, 19, 32, 42]
Involve	▪ Create methods to collect concerns/ideas of specific populations about a health service/policy ▪ Inform decision makers about specific communities’ values, preferences, and needs regarding broader system-level issues ▪ Inform management at facility about their patients’ values/preferences/needs	▪ Communities previously excluded or oppressed by healthcare structures, from a variety of backgrounds ▪ People whose voices were historically ignored	▪ Patients participated in the development of communication structures through focus groups and surveys ▪ Engaged community groups to learn about facilitating the patients ▪ Created informational material to enhance participants understanding of the issues ▪ Ongoing dialogue through various formats to identify concerns/hopes, and inform decision makers	[20, 23, 28, 29]
Collaborate	▪ Advise on planning of an initiative for a specific population ▪ Advise health authority how to improve services for a particular population	▪ A diverse range of participants, including members of the population targeted by the initiative, and people who were personally or professionally interested in the issue	▪ Created an advisory panel or committee ▪ Participants attended regular meetings ▪ Provided opportunities for participants to learn about the issue ▪ Participants provided information, advice and feedback about the initiative. Through the process the facilitators responded to this input, creating a cycle of feedback and response over time	[22, 24–27, 30]
Empower	▪ Plan and implement a community health promotion initiative	▪ Diverse range of participants, including members of the population targeted by the initiative, and people who were personally or professionally interested.	▪ Created a partnership with the participants ▪ Supported partners to plan activities and evaluation criteria of the project ▪ Provided learning opportunities for participants	[21]

We identified trends on engaging marginalized people and trends within the various categories of engagement. Together they provide information about supporting marginalized populations to: 1) attend patient engagement events; and 2) participate meaningfully in these processes.

### Attending patient engagement events

Patients who participate in patient engagement may experience barriers to participating and incur direct costs (e.g., childcare and transportation) and opportunity costs (e.g., missed social or work opportunities). Such costs increase when patients are involved in extensive engagement experiences. Engagement facilitators may attempt to minimize the costs of participatinghowever, minimizing costs and barriers is not sufficient. Participation costs are relative to an individual’s social location, including, for example, gender, income, and employment status, which in turn shape the resources they can draw from to participate.

Authors noted all engagement processes require time commitment from participants, though amount of time, and other direct and indirect costs of participating, vary across engagement processes. One-time events drew a large number of participants from diverse populations [18, 19], while many people did not participate in or dropped out of engagement processes involving recurring events, citing time and responsibility commitments as barriers [18, 20–22].

Barriers also varied by dimension of marginalization and participant’s life context. Low socioeconomic status seemed to be associated with greater barriers to participation, due to higher opportunity costs on their ability to meet basic needs (e.g., accessing free meals, shelter, and temporary work).

The setting of engagement events also presented barriers. Some participants were physically incapable of getting to events due to disabilities [23]. Others felt uncomfortable in formal and unfamiliar spaces, such as academic setting [24]. In other cases, discomfort came from distrust of institutions associated with the location (such as criminal justice systems) [19]. Finally, language was a barrier for many, and interpreter or translator costs often are seldom covered by engagement budgets [25].

A primary strategy to help marginalized people attend engagement events was to reduce the cost of participating by providing childcare, transportation stipends, honoraria, and food [18–21, 25, 26]. Some processes were flexible about meeting locations and times [20–22, 25]. Several projects sought to overcome participation barriers by working with community groups [20, 23, 26, 27] which helped facilitators gain access to marginalized groups and allowed them to learn about the community’s needs and necessary considerations for successful engagement [15].

Some successes in more intense participation came through engagement processes built into interactions between patients and practitioners, particularly in community-based services with recurring interaction with patients [28–31]. In these processes, there were no additional costs to attending an engagement event beyond seeking care and patients could directly benefit from changes to services. The success in engaging vulnerable patients may be due to patients’ trust in the provider and reduction of access barriers however, by relying on current patients for participation, such processes inherently exclude those not already accessing services. Projects focused on engaging women used a variety of strategies to overcome barriers such as providing childcare and having flexible meeting options to accommodate women’s schedules [18, 19, 22, 25, 29].

Patients also need motivation to participate. The literature suggests that people need more motivation to attend processes requiring higher commitment and participation costs. Generally, people are motivated by issues relevant to their lives or the belief they will benefit from participating. As with engagement costs, perceived relevance and value of participation is shaped by social location.

Across all categories of engagement, participants were motivated by opportunities to improve health services for themselves and others [18, 21, 23]. When cost of participation was minimal, such as during consultations, interest in improving the system and having one’s voice heard was sufficient motivation [18, 19]. At increasing levels of engagement however, benefits needed to be more direct to motivate people. Projects identified as being at the levels of collaboration and empowerment, which required intense commitment, tended to have a more direct impact on participants, either through changes to health services or a benefit gained through participating, such as gaining new skills.

Most projects, particularly those involving more intense levels of engagement, engaged participants on issues related to a specific health service. Participants were recruited from the service and patients were motivated to participate because they had opportunity to directly impact these services and therefore their own lives [21, 26, 28–32].

Engagement projects in which outcomes of engagement had a more distant or abstract effect motivated participants with other benefits. These benefits included empowerment and increased confidence [23, 26], meeting new people, gaining skills that could apply elsewhere, including employment [21, 23, 25], reducing isolation, developing community networks and furthering personal growth and employability [21]. However, these benefits may not be sufficient to motivate economically marginalized populations who are struggling to meet basic needs.

### Meaningful participation

Even when people are motivated to participate and to overcome barriers to attending an engagement event, patients experience barriers to communicating their preferences, needs, and values to health service planners. Many of these barriers lie in the location of power in engagement processes, which affects whether certain groups of people participate, how they participate, and how their participation is legitimized as knowledge to inform healthcare decisions. Meaningfully engaging diverse populations therefore begins by specifically addressing power dynamics among various participants and between facilitators and participants.

Power dynamics between facilitators and participants shape how knowledge is generated, how problems are defined, and what ideas inform healthcare decision-making [11, 18]. Participants may not contribute their lived experience as evidence when issues are defined by facilitators instead of patients and “expert” evidence is privileged. Some participants believed facilitators had power over their health care and worried giving negative feedback could negatively affect their care [23]. Others felt facilitators represented mistrusted institutions, leading them to be less honest about their values, preferences, and needs [21] or to be skeptical of the engagement process [23]. Several articles described professionals’ tendency to dominate engagement events through their use of formal language [21] or simply by being present because other participants tended to believe professionals were the most knowledgeable about the subject [22].

There were a variety of ways facilitators supported participants to contribute. Most importantly, meaningful patient engagement was built on a foundation of trust, through which participants felt safe and comfortable to participate. Trust was particularly important in projects focused on sensitive issues, such as mental health and sexual health. Strategies to enhance trust included: limiting the presence and contribution of professionals (including decision makers and participants who are contributing professional opinions) [21, 22]; creating environments that normalize people’s life-context by surrounding them with people who have similar backgrounds and experiences [18, 19, 25]; and providing opportunities for people to build trust with other participants prior to sharing sensitive information [18, 19].

After laying this foundation, engagement processes should reinforce trust and safety while empowering participants. Several common strategies were used, including processes that gave participants the power to name and define issues that were important to them [20, 23, 24], explicitly prioritizing and legitimizing lived experiences as a form of evidence [22, 27], supporting women to contribute in a variety of ways, and acknowledging their contributions [21, 23, 25, 28].

### Findings from interviews and focus groups

Many of the findings from the literature were confirmed and expanded upon through focus groups and interviews with community members and health services planners.

### Attending patient engagement events

Patients confirmed the barriers to participating in engagement due to direct costs of participation, such as time and transportation, and opportunity costs, such as lost social or work opportunities. Women with young children particularly faced challenges, such as cost of transportation for themselves and their children, and challenges making appointments. Providers who worked with marginalized women echoed that many of their clients face challenges organizing schedules and getting to appointments on time.

Participants stated their motivation to engage arose from their perception that they would personally benefit, echoing themes from the literature. In addition, many said they were motivated by giving back to the community and feeling like their voices would be heard:

*“I think for me, my input is very important. My words are important. That’s all I got*.” (Patient participant in focus group).

The importance of being heard and respected was expressed most frequently by marginalized women who had experienced discrimination from some healthcare providers, yet whose lives had been positively impacted by compassionate or empowering healthcare providers. They said monetary incentives were useful and drew their attention, yet remuneration was less important than feeling like they would have an impact on health services. This underscores the importance of patient engagement being authentic and health service planners listening to patients and addressing their concerns as much as possible.

*“In the future, we want a better system for our sons, for our family.”* (Patient participant in focus group).

“*So if we’re actually told that we were going to be heard, and that our opinions mattered, would make us be able to move forward, because a lot of the times, okay, you’ll put it towards us. “Okay, yeah, we want your input,” but that’s about it, right*?” (Patient participant in focus group).

Health services planners held similar views. One informant who worked with Indigenous populations stated, “*if you're going to ask the opinion and engage community, you have to be willing to go to bat for them later to make the change and advocate for the change.*” (Planner for Indigenous health services).

Both planners and patients were clear about the importance of valuing patients’ participation, being clear about how exactly patients voices were contributing to their programming, and doing what they said they said they would do.

Planners also motivated people to participate by providing patients services they were not otherwise able to afford, such as hairdressing. Finally, they found patients to be highly motivated to engage in relation to issues directly impacting their lives, such as changes to health services they receive.

Consistent with the literature, individuals reported feeling uncomfortable approaching new settings, such as new health care providers, due to a lack of trust and confidence, and even fear. These women would likely have a similar reaction to attending patient engagement in an unfamiliar setting. Planners discussed the value of working with community agencies that had relationships with the community in order to increase potential participants’ comfort and decrease their fear in the process. For example, they stated the value of recruiting participants through personal invitations from community providers and holding engagement events in the community agency’s location, where patients are already comfortable:

“*For many of those groups it's about building the relationship with the people who support them in various places and having them do a one-to-one. ...And the personal ask from the providers who knew those women was the only thing that got them there, because then they could authenticate it. They got asked, so it meant that they were important and that their voice was important*.” (Coordinator for Community Health service)

Furthermore, several providers who worked in community health centres described success with engagement by making it part of the organizational culture and conducting ongoing patient engagement, formally and informally, to gain patients’ input in service decisions. Informal engagement enabled providers to gain input from a diversity of voices, not just those individuals who were most interested in participating.

### Meaningful participation

Planners elaborated on the themes of power and the purpose of engagement in relation to how patients express their lived experiences and how planners understand these as evidence in the planning process. They advised caution in deciding on topics of engagement and the way patients are intended to contribute. They felt that by selecting engagement issues, planners might miss the actual issues that were important to patients. By having full control of the agenda, planners may leave little space for patients to share what really matters to them. One informant said they start all engagement activities by asking the community what they want to talk about. Planners also warned e about power imbalances that arise when asking patients to share intimate details of their lives as evidence, while professionals share facts, numbers, and system-level data. They felt engaging patients over issues that matter to them, and empowering them to provide the type of input they want was essential to fostering meaningful participation. Moreover, patients noted the importance of not just having an opportunity to provide input, but of their input leading to a change in the program or service being planned, as a signifier that their engagement was meaningful.

Patients confirmed power imbalances arise when the engagement facilitator is also the person who provides the service in question. Many patient informants expressed discomfort with providing negative feedback and worry about their future access to care if they voice dissatisfaction. Several specified they would want the facilitator to be someone who was neutral and who did not provide them services. For example,

*“If I criticize the way she's doing her job, she's going to look at me a different way and I’m not going to get my… the services that I would be before. Or I'll be considered a mouthpiece and she's just not going to listen to anything I say now.”* (Patient participant in focus group)

“*It's definitely a worry, because I'm already struggling to get the care that I need right now, so if I make things even worse by putting her down or something like that, or it's going to get even worse kind of thing*.” (Patient participant in focus group)

Planners with substantial experience engaging marginalized patients described the importance of engaging patients on issues with which patients had concrete experience. Asking patients to engage with health systems issues or other ideas that are abstract from their experiences places a burden on patients to learn about the issues they were engaging about, which is not only a deterrent but may also diminish the importance of their values, preferences, and lived experience if they are not able to apply them to the issue.

“*You would have to do so much work to make that space available and supportive, and help them culturally translate about this environment they've walked into.*” (Patient engagement facilitator)

Patients confirmed these ideas through the ways they responded to questions about “patient engagement.” Despite our efforts to exemplify it in concrete ways, the concept of “patient engagement” seemed difficult to relate to. When asked how they would want to be engaged, interviewees found it difficult to develop theoretical responses about processes they did not have experience participating in, and instead often responded by discussing their individual healthcare experiences. We interpreted this to mean the concept of patient engagement per se lacked meaning to patients and was difficult to conceptualize.

Conversely, patients felt they could definitely provide input on concrete issues such as their experiences with health services. One informant also suggested she would be interested in observing practitioners and critiquing their interactions with patients.

Both planners and patients highlighted the importance of creating engagement opportunities matching the abilities and preferences of those participating. Some patient informants felt challenged to participate in group discussions, particularly large groups. Others said they did not feel comfortable in group discussion at all, and said they needed another way of participating, such as one-on-one discussions, or writing down comments.

*“[in groups] you might not even say a word because other people… by the time I think of something to say, they've moved on*.*”* (Patient participant in focus group)

Planners with extensive engagement experience provided paper for patients to write down their ideas, or incorporate opportunities for patients to share their ideas individually. They also used group facilitation strategies that enabled everyone to participate. For example,

“*And a healing circle … even the smallest, squeakiest voice can be heard. And so I've learned as well when using the talking stick, that's another powerful, powerful tool when engaging, and it honours the learnings from others”* (Planner for Indigenous health services)

Both patients and planners agreed facilitators need to gain patients’ trust and comfort in order to meaningfully engage them. Facilitators can build trust by holding engagement opportunities in familiar settings and involving trusted agencies as well as by signaling that patients’ participation was valued by providing food and taking care to organize events in ways that suited patients:

*“Makes you feel like what you have to say is important because you guys put the time to make us feel as comfortable by doing these little things. Not that it was necessarily needed or whatever, but it just makes you feel that what you have to say is worthy of going that extra mile and putting this whole thing out, you know.”*(Patient participation in focus group)

Certain groups such as people who use drugs, are Indigenous, or whose gender is non-conforming reported experiencing discrimination in society and from health service providers. To build trust and comfort among these groups, it is essential to actively normalize their experience within the context of the engagement. For example, facilitators may normalize diverse gender identities by starting a session asking people about the personal pronoun they wish to use.

Some patients said the presence of healthcare executives or decision-makers had potential to make them uncomfortable. However, both patients and health service planners said it was not only appropriate for executives and decision-makers to be in the room, it was important for them to hear directly from patients. Yet both groups felt it necessary to neutralize the power of professional participants through symbolic gestures such as only using plain language (no jargon), wearing neutral clothing (“*no suits*”), and ensuring they only spoke when appropriate (e.g., they had permission from patients or patients spoke to them directly).

*“I don't think [having executives present] ever changed the dynamic in terms of like in a bad way. I thought it always was excellent. As long as you can have that person, you are bearing witness from a one-way glass, and the only time you can speak is if we agree that the question in the room is appropriate to be directed towards you and that you can engage back with it, because otherwise we will affect the dynamic in the room*.” (Patient engagement facilitator)

These findings highlight the need to adapt engagement processes to patients, rather than inviting them to participate in a process designed for health service planners.. This requires shifting power to patient participants, such as by engaging them at a location where they hold power, minimizing participation of people who have power over them (e.g., their health care providers), giving them choice in how they will participate, and empowering them to name the issues that are important to them or to criticize the system that has discriminated against them.

Facilitators can best adapt engagement processes by considering participants’ multifaceted identities, including age, gender, culture, education, and work background. The knowledge and skills patient participants possess, as well as their participation preferences, should dictate engagement format and strategies. For example, one informant who worked with Aboriginal communities indicated processes for engaging many Aboriginal communities should adapt to the matriarchal nature of the community, importance attached to elders, and other cultural traditions. On the other hand, when working to engage women who are mothers, the point of interaction will likely be different, and the way of engaging them might also look quite different. Facilitators should also examine their own identities and how these, in relation to patients’ identities, shape power dynamics in engagement processes.

### Development of the model

What emerged from the synthesis of the findings from the literature review, interviews, and focus groups was not a single process for engaging marginalized populations, but instead an iterative process of *fitting* the engagement method to a particular situation (e.g., to patients’ needs and barriers to engagement, type of input patients are contributing, ability of the program to act on patients’ input, and health service planners’ needs). The model is intended to help planners develop an engagement process in which participants feel safe and empowered to share their lived experience as a form of evidence in health service planning. Underlying this iterative process is a set of principles for meaningfully engaging marginalized people in health services planning.

Figure 1 shows the first iteration of the model, which starts with an assessment of program leadership’s readiness to listen to the input of marginalized populations and use it to inform their planning process. If the program is not prepared to engage patients in a meaningful and authentic way, it should not proceed with patient engagement. Next, program planners engage in an iterative process of defining a clear purpose for engagement, identifying population(s) of interest, determining an appropriate environment for engagement, developing a process of information sharing, and considering benefits to those being engaged. These elements are viewed through a gender lens that recognizes how gender shapes experiences, identities, and opportunities. The IAP2 spectrum of participation, which is represented as a funnel, is used as a framework to determine an appropriate method for engagement. From there, consideration is given to how to recruit participants beyond those who already seek healthcare, conduct engagement that empowers marginalized patients to engage in meaningful ways, then feed patients’ input into the decision making process, followed by feeding information back to participants on how their input was used to inform decisions. Finally, processes and outcomes of the engagement are evaluated. This model was used in two pilots and refinements were made based on those experiences. The refined model is shown in Fig. 2.

In our refined model, we shifted determination of an appropriate engagement environment (setting) to follow the choice of engagement method, as part of a new logistics category, which also incorporates recruitment and barriers to engagement. This shift is the most significant aspect of the revised model because the choice of engagement level and method dictate considerations for all other aspects of the process.

For the engagement itself, considerations for facilitation and power inequities were highlighted due to the importance they had in the pilots. As well, some changes to the graphic design were made in order to better communicate concepts in the model, such as placing principles^1^ on a circle surrounding the rest of the model to indicate they are important throughout all stages.

The experiences from the pilots were also used to refine the planning manual. Questions were clarified and reordered based on feedback from planners who participated in the pilots. (The final tools can be accessed from the Canadian Foundation for Healthcare Improvement’s (CFHI) Patient Engagement Resource Hub [33]^2^).

## Discussion

Recognizing that most engagement processes are gender-blind, the purpose of this study was to develop a model to engage marginalized women in health services planning. We drew on literature and qualitative interviews and focus groups with participants from diverse populations and with health services planners and researchers with experience engaging marginalized populations to create a planning model. This model differs from traditional methods of patient engagement, such as those that “activate” patients and deploy them into planning processes. Such methods often put the onus on patients to learn to interact with health services planners in ways that conform to health service planners’ norms and reinforce existing power dynamics. Instead, our model puts the onus on those planning health services to listen and respond to voices of patients who have not traditionally been engaged and to engage with them in the ways in which these participants prefer to engage. This model calls attention to power dynamics in engagement processes and promotes strategies to empower patient participants to ensure they can convey their preferences, values, and needs to healthcare planners.

A recent study by Bellows et al. [34] explored perceptions of patients, providers, and leaders involved in patient engagement at a large health authority in Canada about what they believe makes for successful engagement. Though the study was gender-blind and did not address issues of diversity or marginalization, it did find similar themes to ours, including the importance of having a clear purpose, avoiding jargon, and evaluating the engagement. Interestingly, while patients in this study noted some demographic groups were “missing” from patient engagement experiences, evidence from the article suggests the patient engagement experiences were not necessarily supportive of marginalized groups. For example, in that study, leaders articulated the importance of “seeking patient advisers who have dealt with and accepted their healthcare experience and who are, therefore, emotionally ready to participate” (p. 23), which suggests they expect patients to conform to their expectations of appearing unemotional; thus they would exclude individuals who express themselves in emotional ways. Moreover, by requiring that patients had “dealt with and accepted” their healthcare experience, they suggest they would exclude those individuals who, for example, have been discriminated against by the healthcare system and had not “accepted” this. They also stated that leaders expected patients to be “active participants” versus passive ones who “*just show-up once a month and eat their sandwiches*” (p. 21) with no consideration that engagement methods may silence some participants.

There is growing recognition that health services planners need to create more inclusive and accessible patient engagement experiences in order to meaningfully engage a diverse range of populations [35]. In another recent paper, Ocloo and Matthews [36] criticize existing models of patient and public involvement as “too often rooted in a mechanistic, controlled and professionally dominated approach” (p. 629) and identify many of the same barriers to patient engagement as we identified in our research. They suggest “broader frameworks and methods of involvement should be used that offer better ways to share power with healthcare professionals. Central goals of involvement should focus on issues of inclusivity and representation, equalities, non-discrimination and empowerment” [36](p. 629). The model we present seeks to provide just such a framework.

In addition, there is a growing interest in engaging patients in planning and conducting health research [37, 38] and the importance of inclusiveness in this endeavor has been raised [39]. Further, lack of training in patient engagement for researchers, healthcare partners, and other stakeholders has been noted [40]. The principles and process of engaging marginalized populations presented in our model could be applied to research and the material produced in our project may be useful to researchers as well as healthcare planners.

### Limitations

The literature review was limited by the difficulty of finding relevant literature, resulting from the emerging nature of the field, and variations in terms and definitions surrounding patient engagement. Further, patient engagement is often conducted in institutions that either do not focus on publishing their processes (such as community-based or government institutions) or do not make publications continuously and readily accessible. The terms vary, and many reports are no longer accessible because of the continuously changing nature of government and non-governmental organizations and their websites.

The interviews and focus groups engaged patients who we were able to recruit. Given the focus of our project on marginalized populations, we made significant efforts to be inclusive in our recruitment; for example, in our pilot of the model with the Public Health Nursing home visit program, rather than recruiting women who were receiving healthcare services, we recruited women through community-based organizations that provide services outside the narrowly defined “health services”, including a local high school, a First Nation, a group for post-partum mothers, and an organization serving refugees. These organizations have developed their services in a more community-based way than healthcare organizations and consequently serve a broader population of marginalized people who may stay away from healthcare services. However, we recognized we likely missed the most marginalized individuals, who are not connected to any services.

Although the initial model was tested with two pilot projects in order to refine the final model, further work is needed to test the robustness of the model by applying it to more programs, including the engagement of patients in planning and conducting research.

## Conclusions

Using a literature review and qualitative interviews and focus groups, we created a model to assist health services planners to plan, implement, and evaluate meaningful patient engagement with patients not traditionally engaged in healthcare planning. In addition, we produced both printed and online training materials to assist health system planners to use the model. Starting with a concern to improve participation of marginalized women in healthcare planning, we developed a model which reminds planners to consider how gender intersects with other aspects of social location and identity to limit participation of various groups, including gender non-conforming individuals (e.g., LGBTQI, two spirited), non-English-speaking individuals, and those with experiences of substance use, low income, violence, homelessness, and/or mental health challenges. This model emphasizes barriers to participation related to these intersections and provides practical guidance to reducing barriers. Moreover, it puts responsibility for learning how to listen and respond to voices of patients who have not traditionally been engaged on those planning health services rather than requiring patients to behave in ways preferred by health service planners. Further research is needed to explore the effectiveness of this model in the meaningful engagement of marginalized populations healthcare planning and research.

## Additional files

Additional file 1: Contains the guides for semi-structured interviews and focus groups with female community members. (PDF 86 kb)

Additional file 2: Contains guide for the semi-structured interviews with health service planners and researchers who had experience engaging patients, particularly people whose voices have not traditionally been heard in health services planning. (PDF 69 kb)

## Abbreviations

CFHI: Canadian Foundation for Healthcare Improvement
CIHR: Canadian Institutes for Heath Research
CINHAL: Cumulative Index to Nursing and Allied Health Literature
IAP2: International Association of Public Participation
LGBTQI: Lesbian, gay, bisexual, transgender, queer or questioning, and intersex
MEDLINE ®: Medical Literature Analysis and Retrieval System Online

## Footnotes

1 The principles of the model are: gender-sensitive; focus on power; recognize and work with diversity; recognize the needs of decision makers in the process; value lived experiences; commit to using the patients’ input; and report back to patients.

2 More recently, the material was adapted to create an online learning module to assist health services planners to conduct meaningful patient engagement with marginalized populations. This course can be accessed online [41].
