# Supplementary material for: Heard and valued: the development of a model to meaningfully engage marginalized populations in health services planning
Source: BMC Health Serv Res. 2018 Mar 15;18:181. doi: 10.1186/s12913-018-2969-1 (PMC5856315; doi:10.1186/s12913-018-2969-1)
Supplement: Supplementary file 1 — Contains the guides for semi-structured interviews and focus groups with female community members. (PDF 86 kb) [file 12913_2018_2969_MOESM1_ESM.pdf]

SEMI-STRUCTURED INTERVIEW SCRIPT – PATIENT ENGAGEMENT

Introduction

Good morning/afternoon! First, I would like to thank you for taking the time to participate in our research project. This study will help us learn about the best ways to include women in planning health care programs so that these programs meet your needs. Today we will be talking about your health care experiences and exploring ways that you might want to be involved in making decisions about health care services. Your contributions will help us to develop a process that we can use to regularly include women in decision making, so that they can have a say in their health care.

Your participation and opinions are important! There are no right or wrong answers and I hope you feel free to say whatever you want to say.

Our discussion is being audio recorded because we want to make sure we capture all of your ideas. The audiorecordings, as well as any notes we make, will be kept secure.

Do you have any questions before we begin?

Questions:

1. Please tell me a little bit about yourself.
   a. *This question is meant to get the discussion going and to elicit a bit about their background to put the interview into context.*
2. Tell me about your experiences with the health care system. This can be, for example, with hospitals, public health units, community health care centres, residential care facilities, or family doctors.
   a. Have any of your experiences been good ones? What made them good experiences?
   b. Have any of your experiences been bad ones? What made them bad experiences?
3. We are interested in finding ways to involve clients of the health care system, like you, in making decisions about health care programs. Is this something you think is worthwhile?
   a. Why or why not?
4. What types of things can health care people learn from patients, like you, to improve health services?
5. How would you want to be involved in teaching it to them?
6. What challenges do you think there would be for becoming involved in this? What could we do to overcome the challenge of [specific challenge elicited if first part of question]?
7. What would motivate you to be involved in this process?
8. If you were involved in this process, what would make you think that it was worth your time?

9. Would you want to be involved on an ongoing basis, or would you prefer to participate in just one event?
10. Do you have any other ideas/thoughts/comments you would like to share about making decisions in health care?

Wrap-up

That wraps up our interview. Thank you for your time, it has been very valuable to us and we have learned a lot from you.
We will give you your Honoraria now. For accounting reasons we need you to sign this receipt.
The next thing we will do is have this interview transcribed and then we will make summaries around themes that we think are important. After that would like to give you the opportunity to look at the way that we interpreted the interview and tell us if you agree. Whether or not you decide to do this is entirely up to you. You don't have to decide now, but if you think that you might be interested we will get your address so that we can deliver the papers to you.
After we have performed and analyzed all of our interviews we are going to write up a report, which will be used to guide the second stage of our project. Would you like a copy of the final report? Can you provide us with an email address or an address so that we can deliver it to you?

You have our contact information on the consent form please feel free to contact us for any reason.

Thanks again!

Semi-Structured Focus Group Script – Patient Engagement

Introduction

Good morning/afternoon! First, I would like to thank you for taking the time to participate in our research project. This study will help us learn about the best ways to include women in planning health care programs so that these programs meet your needs. Today we will be talking about your health care experiences and exploring ways that you might want to be involved in making decisions about health care services. Your contributions will help us to develop a process that we can use to regularly include women in decision making, so that they can have a say in their health care.

Your participation and opinions are important! There are no right or wrong answers and I hope you feel free to say whatever you want to say.

Our discussion is being audio recorded because we want to make sure we capture all of your ideas. The recordings, as well as any notes we make, will be kept secure.

We have a few ground rules that we want to start off with:

- Everyone's voice is important: we want everyone to have the chance to tell us about your experiences and ideas.
- Open to all ideas: there are no right or wrong answers
- Respect each other's privacy: we will be talking about personal issues. To respect each other's privacy we will not discuss the things that we hear outside of this group.

Do you have any questions before we begin?

Questions:

1. First, we would like to get to know everyone, so we'll go around in a circle and introduce ourselves, and tell us about a time that you used health care and describe one thing that they did that made your feel comfortable or uncomfortable?.
    a. Can you describe any good healthcare experiences? What made them good experiences?
    b. Can you describe any bad healthcare experience? What made them bad experiences?
2. What types of things can health care people learn from patients, like you, to improve health services?
3. How would you want to be involved in teaching it to them?
4. What challenges do you think there would be for becoming involved in this? What could we do to overcome the challenge of [specific challenge elicited if first part of question]?
5. What would make you want to be involved in this process?
6. If you were involved in this process, what would make you think that it was worth your time?

Wrap-up

That wraps up our focus group. Thank you for your time, it has been very valuable to us and we have learned a lot from you.
We will give you your Honoraria now. For accounting reasons we need you to sign this receipt.
The next thing we will do is have this interview transcribed and then we will make summaries around themes that we think are important. After that would like to give you the opportunity to look at the way that we interpreted the interview and tell us if you agree. Whether or not you decide to do this is entirely up to you. You don't have to decide now, but if you think that you might be interested let us know and we will contact you when these analyses are ready.
After we have performed and analyzed all of our interviews we are going to write up a report, which will be used to guide the second stage of our project. Would you like a copy of the final report?
You have our contact information on the consent form please feel free to contact us for any reason.

Thanks again!
