# Supplementary material for: Heard and valued: the development of a model to meaningfully engage marginalized populations in health services planning
Source: BMC Health Serv Res. 2018 Mar 15;18:181. doi: 10.1186/s12913-018-2969-1 (PMC5856315; doi:10.1186/s12913-018-2969-1)
Supplement: Supplementary file 2 — Contains guide for the semi-structured interviews with health service planners and researchers who had experience engaging patients, particularly people whose voices have not traditionally been heard in health services planning. (PDF 69 kb) [file 12913_2018_2969_MOESM2_ESM.pdf]

SEMI-STRUCTURED INTERVIEW SCRIPT FOR DECISION MAKERS

Good morning/afternoon! First, I would like to thank you for taking the time to participate in our research project. This study will help us learn about the best ways to engage marginalized women in planning health care programs to meet their needs. Today we will be talking about your experiences with patient engagement, specifically, the reasons you use patient engagement, the methods you used to engage patients, and how patient engagement affected your programs and policies. We also want to talk about engaging marginalized women in future program planning and your highest aspirations for patient engagement during this planning. Your contributions will help us to develop a mechanism by which we can regularly engage with marginalized women so that they can have a say in how health care programs are developed and implemented.

Your participation and opinions are important! There are no right or wrong answers and I hope you feel free to say whatever you want to say.

Our discussion is being audio recorded because we want to make sure we capture all of your ideas. The audio recording, as well as any notes we make, will be kept secure and all data will be kept anonymous in any reports we write up from this research.

Do you have any questions before we begin?

<u>Context</u>:

1. Please tell me a little bit about your program.
   [This question is meant to get the discussion going and to elicit a bit about their program to put the interview into context.]

<u>Discovery:</u>

2. What does the term 'patient engagement' mean to you?

For the remainder of our discussion, we'd like you to think about engaging patients in the decisions that you make about programs, and not about one-on-one engagement of patients in their own personal health care.

3. Have you ever engaged patients in making decisions about your program?
   [If yes, continue to Q4, if no skip to Q13]

<u>If Yes:</u>

4. Why were you interested in engaging patients in making health system-level decisions?
   Probe: What motivated you to do patient engagement?

5. What strategies and methods did you use?
   Probe: How did you choose which patients to engage?
   Probe: How did you engage them?

6. Did you use any strategies to engage a diverse variety of patients?
   Probe: Were you able to engage patients that have challenges to participating in this type of activity (e.g., have disabilities, have low literacy, are new immigrants, etc.)

7. Think back on your experiences with patient engagement and tell me about a moment that was particularly rewarding or successful. What made it successful or rewarding?

8. What were you hoping to get out of patient engagement?

9. How did the patient engagement affect the program/policy you were engaging with them about?

10. Throughout your experiences with patient engagement, were there particular challenges that you had to overcome? How did you address these challenges?

11. Are there other potential obstacles or barriers to effective patient engagement that you can foresee? How do you think these can be addressed?

12. What lessons did you learn about patient engagement from your experience?

<u>If no:</u>

13. What interests you about engaging patients in making health system-level decisions?
    Probe: What motivates you to do patient engagement?

14. Are there potential obstacles or barriers to effective patient engagement that you can foresee? How do you think these can be addressed?

<u>For everyone:</u>

15. Are there scenarios where you think it would be useful to engage patients whose voices typically aren't heard? Why?
    Probe: low socioeconomic status, new immigrants and refugees, etc.
    Probe: We've been thinking about two scenarios of engaging marginalized women in program planning. One would be engaging them in a program that is specifically targeted for that subpopulation (e.g., outreach services for the homeless), whereas the other is engaging patients whose voices typically aren't heard in planning universal services to make those services work better for that population (e.g., engaging homeless people about emergency room services).

16. How would you engage these patients?

<u>Dream:</u>

17. What are your highest aspirations for patient engagement as a whole?
    Probe: In an ideal world, what would patient engagement look like?

Design:

18. Thinking about both your experiences with patient engagement and your aspirations for patient engagement, describe components that you think would contribute to an 'ideal' patient engagement model?

19. How could an effective patient engagement model assist you in your work?

20. Thinking about those 'ideal' components just discussed, will components of this patient engagement model help you make a decision? If so, how?

21. What support or resources would you need to conduct effective patient engagement?

22. We are going to create a patient engagement model. How should we judge if it works?

23. What will inspire you to commit your efforts to patient engagement or to encourage others to adopt and implement a patient engagement model?

24. Do you have any other questions, comments, additions, or stories to share?

Wrap-up

That wraps up our interview. Thank you for your time and for sharing with us. It has been very valuable to us and we have learned a lot from you.

The next steps for our research will be to analyze the data we are gathering from interviews with you and other health care and community agency decision makers, as well as interviews with patients. From all of these data, along with a literature review, we will be creating a patient engagement mechanism that we will then be looking to pilot. Would you be interested in piloting and evaluating our mechanism in a program or policy decision that you are making? (The timeframe will be sometime in the fall or winter).

Thanks again!
